# Relationship between anthropometric measures and early electrocardiographic changes in obese rats

**DOI:** 10.1186/1756-0500-7-931

**Published:** 2014-12-18

**Authors:** Steve Kyende Mutiso, Dennis Kipkemoi Rono, Frederick Bukachi

**Affiliations:** Department of Medical Physiology, University of Nairobi, P.O. Box 30197–00100, Nairobi, Kenya

**Keywords:** Obesity, Electrocardiography, Anthropometrics

## Abstract

**Background:**

The degree of cardiovascular function impairment parallels the degree of obesity and obese subjects have abnormal changes on the electrocardiogram (ECG). Early ECG changes in obesity have not been previously studied. The objective of the present study was to determine the early ECG changes in obese rats and their relationship with anthropometric measurements.

**Results:**

At seven weeks all rats in the experiment were obese and in sinus rhythm. In the experiment resting heart rate was increased (364 ± 13 vs. 313 ± 12 bpm, *P* <0.01). In contrast, the following parameters were shortened: QRS duration (77 ± 3.6 vs. 65 ± 2.6 ms, *P* < 0.01); QT interval (102 ± 5.2 vs. 88 ± 3.7 ms, *P* < 0.05); Q wave amplitude (−12.8 ± 1.0 vs. -5.1 ± 0.9 μv, *P <0.01*); and T wave amplitude (*18*.8 ± 1.4 vs. 5.8 ± 0.6, *P <0.01*). All other ECG parameters remained unchanged. With increased weight the resting heart rate (*r* =0.46, *P* < 0.01) and R wave amplitude (*r* = 0.60, *P* < 0.01) increased.

**Conclusion:**

Early in obesity there are no rhythm disturbances, but resting heart rate is increased. The QRS duration is shortened and Q and T-wave amplitudes reduced signifying ventricular changes related to impaired myocardial depolarization and repolarization. Furthermore, weight gain is correlated with an increase in heart rate and accentuation of the R wave amplitude.

## Background

Obesity is a growing problem worldwide especially due to dietary malpractice and physical inactivity. Obesity is considered a prime risk factor for cardiovascular disease (CVD) [1]. It is now acknowledged that obesity is a global epidemic. About 1.6 billion people are overweight, of these 400 million adults and 40 million children are obese worldwide [2]. Obesity levels have risen sharply across the globe with it being considered an independent risk factor for CVD in children, and is moreover associated with reduced life expectancy [3]. The global epidemic of obesity results from a combination of genetic susceptibility, increased availability of high-energy foods and decreased requirement for physical activity in modern society [4].

Hyper caloric diets have previously been shown to induce obesity in rats [5, 6]. Thus, providing an animal model for studying obesity - an important step towards better understanding of the pathophysiology of CVD in obese humans. The use of sucrose solution has also been described in the literature [6, 7]. However the use of a combination of the two is a novel idea.

Obesity has numerous adverse effects on cardiovascular health and these include: hypertension, heart failure, atrial fibrillation, stroke, prothrombotic states, ventricular arrhythmias and venous disease [8]. It has also been associated with the causation of diseases which are major risk factors for CVD, such as type II diabetes and sleep apnoea [8]. These effects of obesity are achieved through the metabolic consequences and pathological effects on cardiovascular function [3].

Changes in various electrocardiographic parameters have previously been observed in obese humans. A low QRS voltage, flattening of the T-wave in the inferolateral leads and left shifts of the P and QRS axes in obese patients was shown as a significant finding by Eisensten et al. [9]. In addition, increased heart rate, PR interval, QRS duration; QTc interval and voltage (R + S or Q wave in leads I, II and III) and the leftward shift in the QRS vector have all been reported with increasing obesity in another study [10]. Obesity also affects P wave dispersion and duration [11]. The change in P wave dispersion may be closely related to the clinical parameters such as BMI [12]. Furthermore, it has been shown that obesity is associated with increasing of the QT interval [13, 14]. The aim of the present study was to induce obesity using a hyper caloric diet and subsequently determine the earliest attendant ECG changes.

## Methods

### Study design

The study was experimental study design with two randomized independent groups within which there were repeated measures.

### Study animals

Forty male Wistar rats were used for the study. The animals were randomly allocated into two equal groups (n = 20), the control and experimental group. They were housed in eight plastic wire meshed cages (30 cm × 15 cm × 12 cm) placed on a 0.75 m raised surface in the labaratory animal house. Wood shavings used as beddings were replaced every 2 days. They were kept in light controlled quarters at a 12 hour light–dark cycle (lights on: 07:00–19:00 h and lights off: 19:00–07:00 h). All experimental procedures on the rats were conducted during the light cycle. The average room temperature was kept at 21 ± 1°C. The control group was fed on the normal rat diet and water *ad libitum* while the experimental group was provided with the high fat (HF) diet and 30% sucrose solution *ad libitum.*

### Induction of obesity

Two pelleted semi purified, nutritionally complete experimental diets were used . Animals in the experimental group were fed on a HF diet consisting of 30% fat, supplemented with 30% sucrose solution and the control with a normal diet consisting of 5% fat, for a period of seven weeks.

The HF diet was freshly prepared and it contained 30 g of fat/100 g of pellets (25 g of vegetable oil and 5 g of soybean oil). To formulate the feed, vegetable oil was added to a commercially available normal pellet food (Unga Feeds Kenya Ltd, Nairobi, Kenya). The vegetable oil was chosen on the basis of its high digestibility (99.7%) [15]. The 30% sucrose solution contained 30 grams of cane sugar dissolved in 100 ml of water. The normal rat diet contains 5 g of soybean oil/100 g of pellets. Because the emphasis in the experiment was on dietary fat, the amount of protein and all of the essential minerals and vitamins required for rats were equalized for the HF and normal diets.

### Determination of BMI

The weight and nose-anus length (NAL) of the rats in the control and experimental groups were measured at the start of the experiment and after seven weeks. BMI was determined by dividing the weight (g) and the square of the nose-anus length (cm). Thus, obesity was defined by a BMI of greater than 0.68 g/cm^2^ as previously described by Novelli et al. [6]. Rats that did not meet the BMI in the experimental group after seven weeks were to be excluded from the study. However, all the rats in the experimental group attained the target BMI and were all included.

### Recording ECG

The ECG record (Lead II) of each animal was taken before and after the seven weeks. The rats were anaesthetized by use of Ketamine (0.2 mg/g bodyweight intraperitionally, IP) before recording the ECG. The ECGs were recorded by use of Power Lab Data acquisition apparatus (Model ML865, ADinstruments, Dunedin, New Zealand). The following standard ECG variables were analyzed: rhythm, heart rate, P wave amplitude and duration, PR interval, QRS duration, QT interval, QTc interval, RR interval, Q, R, S and T wave amplitudes. The QTc interval was derived from the QT interval using the Bazzet’s formula: QTc = QT Interval /√ (RR interval) [16].

### Statistical analysis

Data were analyzed using the two tailed student *t* - test for repeated measures. Pearson correlation analysis was used for estimating the relationship between anthropometric measures and ECG parameters. The data for each group were expressed as mean ± SEM. P value *<* 0.05 was considered statistically significant.

### Ethical considerations

The study protocol was approved by the University of Nairobi Ethics review Committee. The study was conducted in accordance with the internationally accepted principles for laboratory animal use and care [17]. Animals were handled with care and in accordance with the FELASA guidelines [18].

## Results

### General anthropometric characteristics of study animals

The general characteristics of the rats at the beginning and at the end of the study are shown in Table 1. At the beginning of the experiment there were no significant anthropometric differences between the control and experimental groups. However, after seven weeks, there were significant changes in weight (229.2 ± 7.26 *vs*. 314.7 ± 5.78 g, *P* < 0.01), nose-anus length (20.18 ± 0.18 *vs.* 21.14 ± 0.16 cm, *P* <0.01) and BMI (0.56 ± 0.01 *vs*. 0.70 ± 0.01 g/cm^2^, *P* <0.01) between the control and experimental groups.

**Table 1:** **General characteristics at baseline and at seven weeks**

	Control	Experimental	***P***value
	(n = 20)	(n = 20)	
**Weight (g)**			
Baseline	214.7 ± 9.96	216.4 ± 8.59	NS
At seven weeks	229.2 ± 7.26	314.7 ± 5.78	<0.01
**Nose-anus length (cm)**			
Baseline	19.59 ± 0.30	19.17 ± 0.26	NS
At seven weeks	20.18 ± 0.18	21.14 ± 0.16	<0.01
**BMI (g/cm** ^**2**^ **)**			
Baseline	0.55 ± 0.02	0.58 ± 0.01	NS
At seven weeks	0.56 ± 0.01	0.70 ± 0.01	<0.01

Values expressed as mean ± S.E.M. NS, not significant.

**Figure 1:**
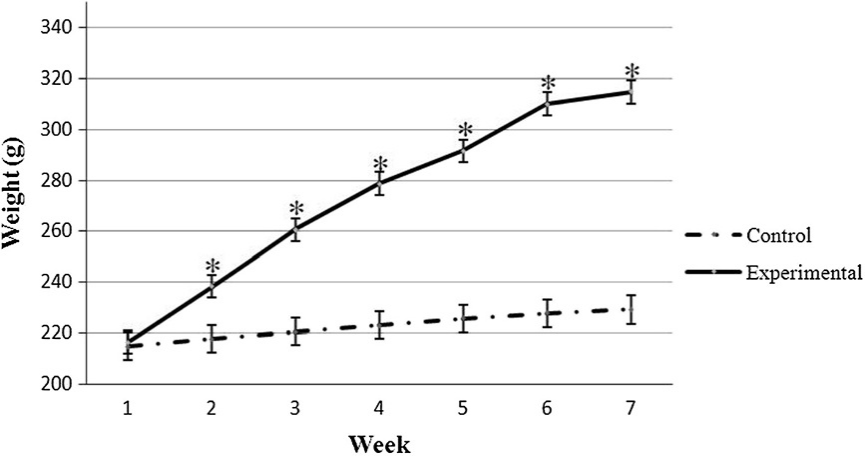
**Weight changes between the two groups over the seven week period.** Error bars represent the S.E.M. *control vs. experimental (*P* < 0.01).

**Figure 2:**
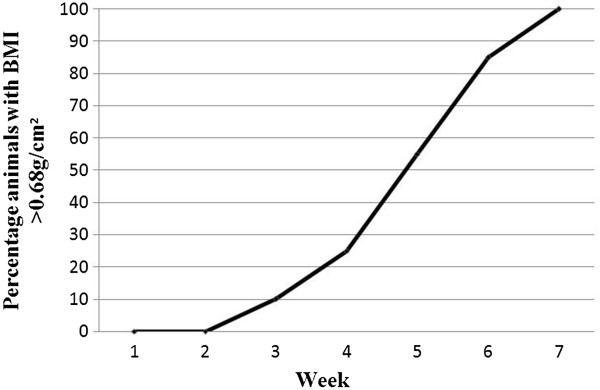
**Percentage rats acquiring a BMI of >0.68 g/cm** ^**2**^ **over the seven week period**.

The progressive changes in BMI in both groups over the seven week period are shown in Figure 1. None of the animals in the control group were obese at week 7. In contrast, the percentages of the rats in the experimental group that attained the greater than 0.68 g/cm^2^ increased sharply after week 2 (Figure 2).

### ECG measurements

**Figure 3:**
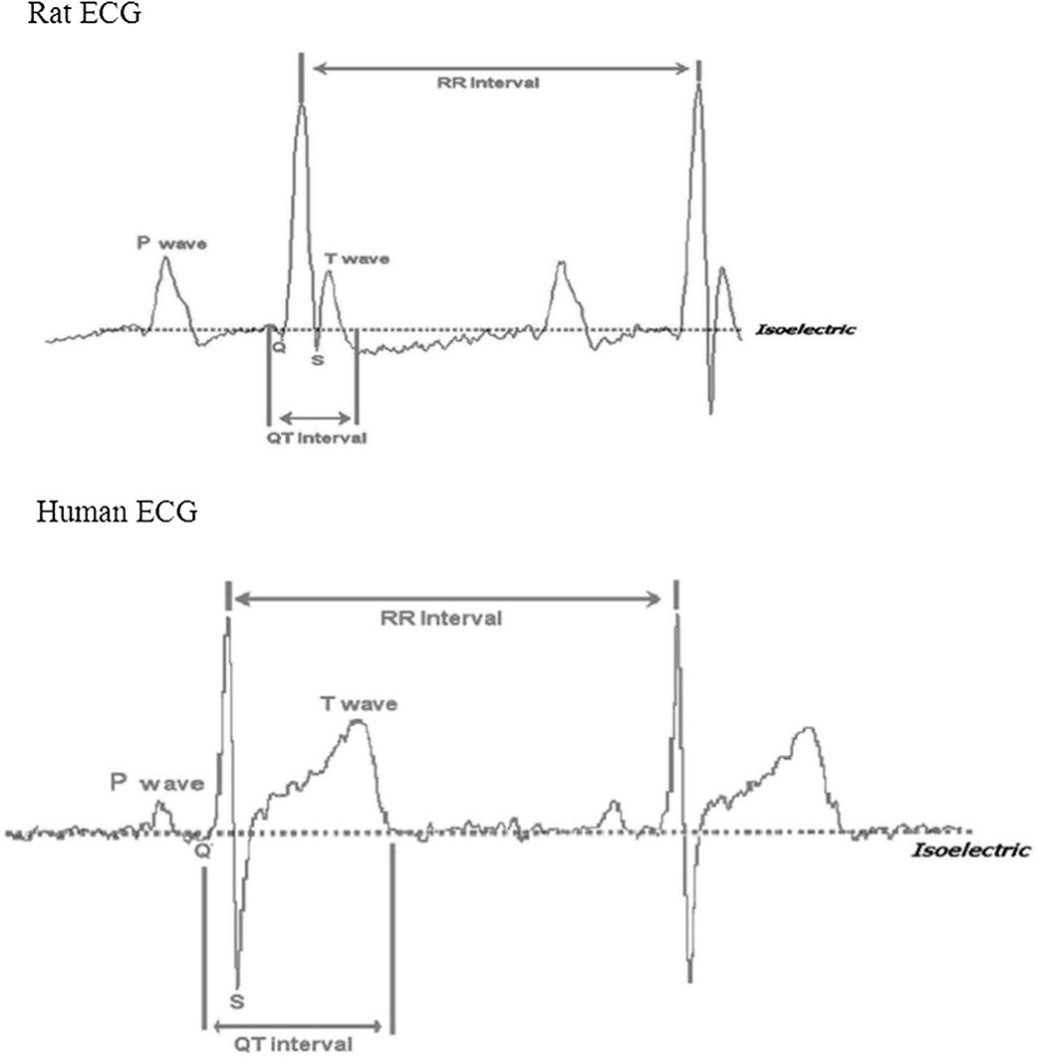
**Normal human and rat ECGs showing the waves and time intervals.** The rat ECG compared to the human ECG has both positive and negative deflections. These deflections, just like in humans, are as a result of action potentials in the myocardial cells. The positive deflections include the P, R and T waves. The negative deflections include the Q and S waves. The difference from the human ECG is the lack of an ST segment.

Rat and human ECGs are similar except for the absence of an ST segment in the former (Figure 3). At baseline ECG parameters were similar in both groups (Table 2). At seven weeks, however, there were significant differences (Table 2): The mean heart rate was significantly higher in the experimental group (313 ± 12 *vs.* 364 ± 13 beats per minute, *P* <0.01). Accordingly, the RR interval was also significantly shorter (198 ± 8.3 *vs.* 170 ± 6.2 ms, *P* <0.01). There were no changes in the P wave amplitude and duration, as well as the PR interval. The QRS duration and the QT interval were significantly shorter in the experimental group compared to the controls (77 ± 3.6 *vs.* 65 ± 2.6 ms, *P* < 0.01) and (102 ± 5.2 *vs.* 88 ± 3.7 ms, *P* < 0.05) respectively. However, the QTc interval was similar in both groups. The Q wave amplitude was significantly shorter in the experimental group (−12.8 ± 1.0 *vs.* -5.1 ± 0.9 μv, *P <0.01*). There were no significant changes observed in the R and S wave amplitudes at seven weeks. However, the T wave amplitude was significantly reduced in the experimental group compared to controls (*18*.8 ± 1.4 *vs.* 5.8 ± 0.6 μv, *P <0.01*).

**Table 2:** **ECG parameters at baseline and at seven weeks**

	Baseline.		Seven weeks.			
	Control	Experimental	***P***value	Control	Experimental	***P***value
**Number Of rats (n)**	20	20		20	20	
**Heart rate**	342 ± 12	357 ± 13	NS	313 ± 13	364 ± 13	<0.01
**RR interval (ms)**	180 ± 7	172 ± 2	NS	198 ± 8.1	170 ± 6.0	<0.01
**P wave amplitude (μv)**	19.8 ± 1.9	22.6 ± 2.5	NS	13.9 ± 1.5	14.6 ± 1.7	NS
**P wave duration (ms)**	28 ± 2.0	24 ± 2.4	NS	25 ± 1.9	23 ± 1.5	NS
**PR interval (ms)**	30 ± 2.0	29 ± 1.3	NS	26 ± 1.9	26 ± 1.9	NS
**QRS duration (ms)**	81 ± 4.1	80 ± 3.3	NS	77 ± 3.6	65 ± 2.6	<0.01
**QT interval (ms)**	105 ± 3.6	101 ± 2.0	NS	102 ± 5.2	88 ± 3.7	<0.05
**Q wave amplitude (μv)**	−11.2 ± 1.4	−11.6 ± 1.5	NS	−12.8 ± 1.0	−5.1 ± 0.9	<0.01
**R wave amplitude (μv)**	71.7 ± 9.0	78.3 ± 10.2	NS	61.3 ± 8.4	72.3 ± 8.9	NS
**S wave amplitude (μv)**	−20.8 ± 2.1	−29.1 ± 5.5	NS	−28.4 ± 4.5	−26.6 ± 4.0	NS
**T wave amplitude (μv)**	15.9 ± 1.4	17.9 ± 1.8	NS	18.8 ± 1.4	5.8 ± 0.6	<0.01

**Figure 4:**
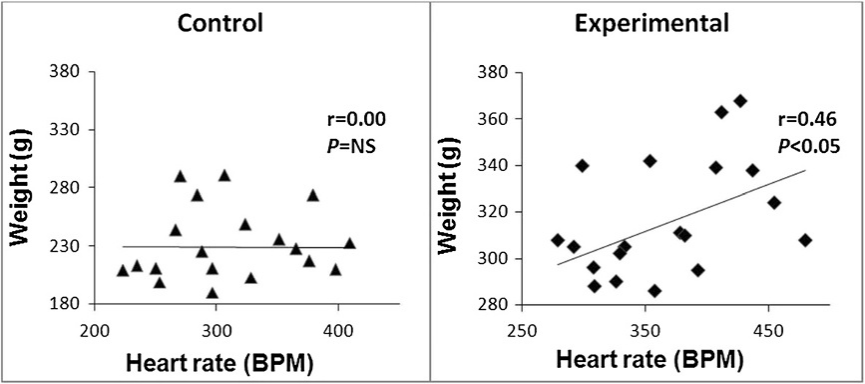
**Scatter plots showing correlations between weight and heart rate between the control and experimental groups at the end of seven weeks.** NS, Not significant.

**Figure 5:**
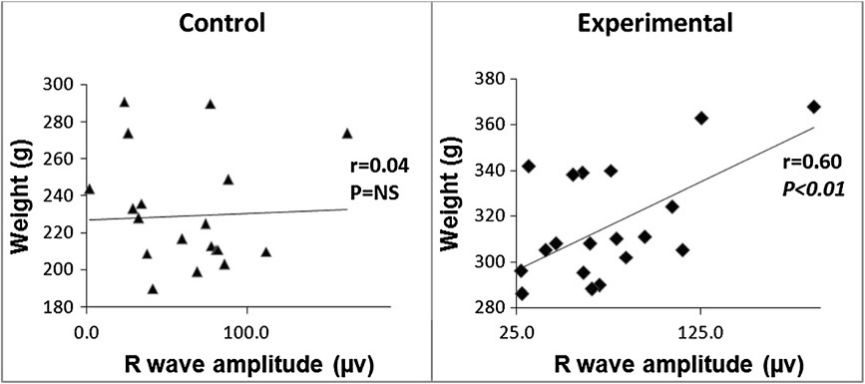
**Scatter plots showing correlations between weight and R wave amplitude at the end of seven weeks.** NS, Not significant.

With bivariate analysis, at the end of seven weeks positive correlations were observed in the obese group between weight and heart rate (r = 0.46, *P* < 0.01) and weight and R wave amplitude (r = 0.60, *P* < 0.01).On the other hand, a negative correlation observed was between weight and RR interval (r = −0.45, *P* < 0.05). These correlations were not observed in the control group (Figures 4 and 5).

## Discussion

Obesity is a growing global pandemic, associated with poor diet and lack of physical exercise. In the long term, it is a major risk factor for CVD. Thus, evaluation of the cardiovascular system is essential in all obese persons. Specific ECG changes have previously been reported in obese humans without clinical symptoms [10]. But there is paucity of data in the literature regarding the progression of ECG changes with increasing weight, particularly the earliest alterations in obesity. We used an animal model to elucidate these changes.

The present study induced obesity in rats by using a hypercaloric diet - a high fat diet augmented with sucrose solution. Hypercaloric diets have previously been shown to induce obesity in rats [5, 6]. It should, however, be noted that this dietary combination is novel and led to rapid weight gain in the experimental animals. Consequently, a number of changes in anthropometric measurements were observed in these animals, namely an increase in weight, nose anus length and the BMI. Similar changes have been recently described [6, 7]. Thus, BMI in rats is a simple and easily reproducible anthropometric measure of obesity.

Following induction of obesity, changes in various ECG parameters were observed. These included, increased heart rate, shortened QRS duration, and reduced Q and T wave amplitudes. Some of these changes have previously been described in humans with established obesity especially increased heart rate ([10, 19]) and flattening of the T wave in the inferolateral leads ([9, 20]). However, the shortening of the QRS duration and Q wave amplitude observed in our study have previously not been reported. The QRS duration, which represents the period of myocardial depolarization, is consistently prolonged in established obesity in humans [10]. The decrease observed in the present study may be attributed to increased conduction rate of the impulse in the myocardium that is an accompaniment of higher resting heart rates seen in obesity. This may also be a result of a reduced ventricular refractory period and a higher velocity of ventricular contraction. The Q wave amplitude was decreased in the obese group. Although Q waves have previously been described in healthy rat ECGs [21], no study has reported them in lead II. The present study, therefore, becomes the first to describe Q waves in lead II of the ECG in healthy rats. The Q wave represents movement of the activation impulse through the septum and its decrease implies that the orientation of the septum is changing to a leftward position. This anatomical shift in septal orientation is expected in obese rats in whom the heart lies more horizontally in the chest due to increased abdominal adiposity. Similar anthropometric changes have been observed in humans [21].

Obesity in humans leads to changes in various anthropometric measurements. These include: increased BMI, waist to hip ratio and waist circumference [22]. Similar changes have recently been reported in obese rats [6] but no previous study has made an attempt to correlate these anthropometric measurements with ECG parameters. Thus, the present study becomes the first to examine this relationship. Increased weight had a positive correlation with resting heart rate. This finding, although expected, is attributable to increased body weight which is associated with increased adiposity [20]. The latter leads to a positive energy balance leading to hyperinsulinaemia and the production of excessive leptin by the adipose tissue. Consequently, this leads to hyperleptinaemia. Both hyperinsulinaemia and hyperleptinaemia lead to the activation of the sympathetic nervous system which results in increased resting heart rate [20]. This appears to be one of the earliest changes in obesity. A positive correlation between weight and the R wave amplitude was only observed in the obese group. The R wave amplitude represents ventricular activity and an increase in the amplitude may denote an increase in ventricular myocardial size. The present study did not examine changes in myocardial mass with increasing obesity. Established obesity however, has been associated with ventricular chamber dilatation due to increased cardiac workload [3]. The dilatation leads to increased wall stress, which predisposes to an increase in myocardial mass and ultimately to eccentric LV hypertrophy [3]. The positive correlation denotes that as the weight of an obese individual increases, the ventricular myocardial size increases to compensate for increased cardiac workload.

Our study was not without limitations. First, the ideal method to record ECGs in rats is without sedation as previously described by Mitchell and colleagues [23]. Ketamine has been shown to increase resting heart rate but its use in both groups provides to cancel out this effect. Moreover, anaesthetic agents have continued to be used successfully in small animal ECG studies. Secondly, the technical limitation associated with lack of equipment to record 12 lead ECG in rats limits the scope of the data obtained including determination of changes in the mean electrical axes. Thirdly, the study did not record ECGs throughout the study period but just at the start and the end. This was attributed to the study protocol where only two readings were to be taken to compare obese and non-obese groups. However, the authors intend to study the progression of ECGs from normal weight to obese subjects in a future study.

## Conclusion

Early in obesity there are no rhythm disturbances, but resting heart rate is increased. The QRS duration is shortened and Q and T-wave amplitudes reduced signifying ventricular changes related to impaired myocardial depolarization and repolarization as well as changes in cardiac morphology. Furthermore, weight gain is correlated with an increase in heart rate and accentuation of the R wave amplitude.

## Abbreviations

BMI: Body mass index
CVD: Cardiovascular disease
ECG: Electrocardiogram
FELASA: Federation of European Laboratories Animal Science Associations
HF: High fat
NAL: Nose-anus length
SEM: Standard error of the mean.
